# Ectopic shoot meristem generation in monocotyledonous *rpk1* mutants is linked to SAM loss and altered seedling morphology

**DOI:** 10.1186/s12870-015-0556-8

**Published:** 2015-07-07

**Authors:** Birgit S. Fiesselmann, Miriam Luichtl, Xiaomeng Yang, Michaela Matthes, Ottilie Peis, Ramon A. Torres-Ruiz

**Affiliations:** Lehrstuhl für Genetik, Technische Universität München, Wissenschaftszentrum Weihenstephan, Emil-Ramann-Str. 8, D-85354 Freising, Germany; Lehrstuhl für Pflanzenzüchtung, Technische Universität München, Wissenschaftszentrum Weihenstephan, Liesel-Beckmann-Str. 2, D-85354 Freising, Germany

**Keywords:** RPK1, *Arabidopsis*, Shoot meristem, SAM, Cotyledon, Monocot, Dicot, Plant embryo, Angiosperm evolution

## Abstract

**Background:**

In dicot *Arabidopsis thaliana* embryos two cotyledons develop largely autonomously from the shoot apical meristem (SAM). Recessive mutations in the *Arabidopsis* receptor-like kinase *RPK1* lead to monocotyledonous seedlings, with low (10 %) penetrance due to complex functional redundancy. In strong *rpk1* alleles, about 10 % of these (i. e. 1 % of all homozygotes) did not develop a SAM. We wondered whether *RPK1* might also control SAM gene expression and SAM generation in addition to its known stochastic impact on cell division and PINFORMED1 (PIN1) polarity in the epidermis.

**Results:**

SAM-less seedlings developed a simple morphology with a straight and continuous hypocotyl-cotyledon structure lacking a recognizable epicotyl. According to *rpk1*’s auxin-related PIN1 defect, the seedlings displayed defects in the vascular tissue. Surprisingly, SAM-less seedlings variably expressed essential SAM specific genes along the hypocotyl-cotyledon structure up into the cotyledon lamina. Few were even capable of developing an ectopic shoot meristem (eSM) on top of the cotyledon.

**Conclusions:**

The results highlight the developmental autonomy of the SAM vs. cotyledons and suggest that the primary *rpk1* defect does not lie in the seedling’s ability to express SAM genes or to develop a shoot meristem. Rather, *rpk1’s* known defects in cell division and auxin homeostasis, by disturbed PIN1 polarity, impact on SAM and organ generation. In early embryo stages this failure generates a simplified monocotyledonous morphology. Once generated, this likely entails a loss of positional information that in turn affects the spatiotemporal development of the SAM. SAM-bearing and SAM-less monocotyledonous phenotypes show morphological similarities either to real monocots or to dicot species, which only develop one cotyledon. The specific cotyledon defect in *rpk1* mutants thus sheds light upon the developmental implications of the transition from two cotyledons to one.

**Electronic supplementary material:**

The online version of this article (doi:10.1186/s12870-015-0556-8) contains supplementary material, which is available to authorized users.

## Background

As typical representatives of dicot angiosperms, *Arabidopsis thaliana* seedlings display a body plan beginning with an epicotyl region harbouring the shoot apical meristem (SAM), flanked by two cotyledons and followed by the hypocotyl, which ends in a root tip carrying the root apical meristem (RAM) [1]. The initiation of cotyledons vs. SAM is largely independent, as evidenced by mutations that delete the SAM but not the cotyledons [2, 3] and vice versa [4, 5].

Although exceptions from normal cotyledon number in angiosperms are known in several genera [6] cotyledon number is a relatively constant pattern element. Modern taxonomy recognizes eudicots with two cotyledons and monocots with one cotyledon, as monophyletic groups [7, 8]. However, the mechanisms of “counting“and arranging these organs together with the SAM in order to establish the apical region are poorly understood.

The use of *Arabidopsis thaliana* mutants with cotyledon defects helps to get a deeper insight into this developmental process. Careful categorization of known mutants displaying cotyledon defects reveals a group, which obviously reflects more fundamental perturbations such as cell differentiation in *altered meristem program* [9, 10], control of meristem cell fate and lateral organ development in *dornröschen* [11] and division plane orientation in *fass* [12]. This leaves a number of seedling mutants whose defects are cotyledon specific. These mutants are regularly linked to defects in auxin synthesis and transport by the polar auxin efflux carrier PIN1, which generates auxin maxima required to induce cotyledon primordia [13, 14]. For instance, mutants of the AGC kinase *PINOID* (*PID*) and D-myo-inositol-3-phosphate synthase (MIPS) frequently produce abnormal supernumerary cotyledon numbers [15, 16] whereas combinations of *pinoid (pid)* with mutants of related kinases, auxin-synthesis genes and the *NPH3*-like gene *ENHANCER OF PINOID* (*ENP/enp*) result in cotyledon-less seedlings which retain a functional SAM [4, 5, 17–19]. In contrast, mutants specifically segregating a monocotyledonous phenotype are relatively rare and known from *sic* mutants in pea and mutations in the *Arabidopsis* receptor-like kinase *RPK1* [20, 21]. The reason for this sparsity is possibly due to redundant gene functions encoded in the *Arabidopsis* genome. In fact, the monocotyledonous phenotype of *rpk1* mutants has a maximum penetrance of ca. 10 % [21, 22], which could be elevated by adding mutations in the related *TOAD2/RPK2*. However, this combination simultaneously resulted in additional severe pattern effects and high frequency of embryo lethality because *TOAD2/RPK2* has adopted additional functions in radial pattern formation [21, 23] and as regulator of meristem development [24].

Avoiding such pleiotropic effects *rpk1-7* and *rpk1-6* single mutants were recently analysed. This revealed that the primary *rpk1* defect stochastically compromises epidermal cell division and PIN1 polarity during embryogenesis [22]. The defect is stochastic because the accuracy of every new cell division depends on whether the redundant *RPK1*-like genes achieve the required threshold of RPK1 function or not. This implies that the *rpk1* defect can become manifest in different stages (time dependence) and in different regions (spatial dependence). The perturbation of epidermal cell division and PIN1 polarity in a cotyledon anlage might disturb or eliminate the establishment of an auxin maximum and lead to monocotyledonous seedlings (henceforth named monocot seedlings for convenience). The existence of SAM-less monocot seedlings suggested an interference with both cotyledon and SAM development during the early globular stage in the strong *rpk1* alleles.

Here we show that SAM-less monocot seedlings retain basic SAM functions. However, they develop a simple morphology with a continuous hypocotyl-cotyledon organization that lacks a clear separation between these structures. The well-developed lamina is sometimes larger than in the wild-type. Although these monocot seedlings have initially no SAM, they have not lost the capacity to generate one. Some develop a delayed SAM or even an ectopic shoot meristem (eSM) on the adaxial side of the cotyledon. Our analyses suggest that the topological peculiarity of these monocot seedlings is linked to the loss of a spatially and timely coordinated expression of SAM specific genes during early embryogenesis, indicating a loss of positional information by altered morphology.

## Results

### Strong *rpk1* alleles generate SAM-less monocot seedlings

**Fig. 1:**
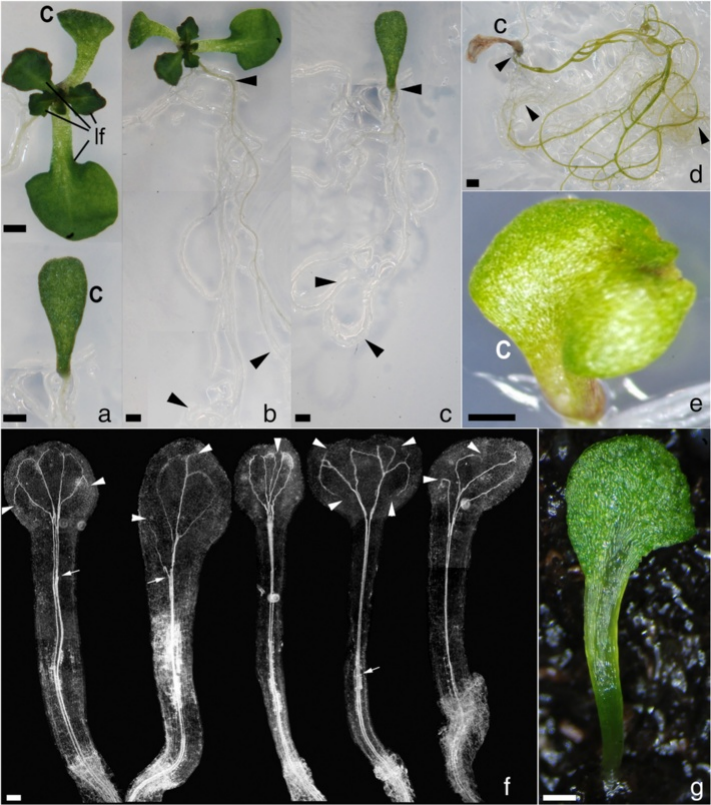
Morphology in SAM-less *rpk1* monocot seedlings. **a** Magnifications of parts of monocot *rpk1-7* seedlings (*gl1/gl1* background) with SAM and primary leaves (top) and without SAM (bottom). **b** and **c** Whole plants with long roots (indicated by arrowheads) illustrate the continuous root growth. **d** A shoot-less monocot seedling from long-term cultivation shows a necrotic cotyledon while the root has continued growth and turned green. **e** A SAM-less monocot seedling with a homozygous *rpk1-7 GL1* background (carrying a *PIN1:GFP* reporter). **f** Seedlings cleared with Hoyers mount visualize the vascular system in the contiguous hypocotyl-cotyledon structure with interruptions (white arrowheads) and supernumerary and/or blindly terminating vascular elements (small arrows). There is no bend recognizable, which in the wild-type separates apically the SAM/epicotyl from the laterally placed cotyledon. **g** A SAM-less monocot seedling originating from the *rpk1-6* allele*.* Cotyledons (c), normal leaf (lf) indicated. Scale bars: 1 mm **a-e**, 0.5 mm **g**, 100 μM **f**

The allele *rpk1-7* was induced in a *gl1* Columbia background and generates ca. 10 % seedlings with cotyledon abnormalities most of them lacking one cotyledon [22]. We detected that, five days after germination, some of the monocot seedlings did not possess developed SAMs in comparison to their monocot siblings (Fig. 1a-c). The cotyledon of these seedlings varied in shape and size and had a well-developed lamina with recognizable adaxial and abaxial sides (Fig. 1). The SAM-less monocots regularly occurred in the pedigree of crosses with plants of different genetic backgrounds with a frequency ranging between 0.5 % and 1.8 % of all seedlings (Table 1). Upon further growing, part of the SAM-less seedlings developed SAMs at some distance from the cotyledon lamina, suggesting that meristem development lagged behind that of SAM-bearing monocots. We considered that the SAM-less phenotype could be a specific character of the *rpk1-7* allele, which is a fast neutron-induced inversion [22]. Therefore, we searched this phenotype in the independently generated *rpk1-6* allele, which is a T-DNA insertion in the RPK1 coding region [22] and found SAM-less seedlings with similar frequencies as in *rpk1-7* (Table 1). The other SAM-less seedlings did never develop a normal SAM but necrotic cotyledons and green, continuously growing roots as long as cultured in sterile 1/2MS medium (Fig. 1d). Notably, in these seedlings the hypocotyl and cotyledon petiole formed a continuous structure without recognizable separation of a SAM region (Fig. 1c, e and f). This was true for both alleles (compare Fig. 1c, e, g) and showed that cell differentiation in these tissues had been fundamentally altered. Whole mount preparations of *rpk1-7* seedlings displayed vascular defects stressing *RPK1*’s link to PIN1 polarity and auxin transport [22]. In *rpk1* monocots, the wild-type diarchic vascular system, which branches into both cotyledons, was variably organized. Either both strands intruded into the remaining cotyledon, or one strand ended in the “hypocotyl“. In other cases supernumerary vascular cell files were formed (Fig. 1f; Additional file 1: Figure S1).

**Table 1:** Frequency of rpk1-7 monocot plants without SAM

*RPK1* mutant line	Wild-types (dicot. *rpk1-x*)		Anisocot./other irregular cots		Monocots + SAM		Monocots -SAM	Back-ground
	+ Trich.^a^	-Trich.	+ Trich.	-Trich.	+Trich.	-Trich.	[%^b^]	
*rpk1-7* allele:								
*FN* ^*9–3*^ *_*1	-	69	-	1	-	9	1 [1.3 %]	*gl1/gl1*
*FN* ^*9–3*^_2	-	290	-	15	-	20	6 [1.8 %]	*gl1/gl1*
*FN* ^*9–3*^_3	-	281	-	12	-	23	2 [0.6 %]	*gl1/gl1*
*FN* ^*9–3*^_4	-	169	-	10	-	35	3 [1.4 %]	*gl1/gl1*
*rpk1-7* allele:								
*FN* ^*9-3*^XPIN1GFP_1	237	-	13	-	18	-	3 [1.1 %]	*GL1/GL1*
*FN* ^*9-3*^XPIN1GFP_2	130	-	6	-	12	-	2 [1.3 %]	*GL1/GL1*
*FN* ^*9-3*^XPIN1GFP_3	171	-	4	-	9	-	1 [0.5 %]	*GL1/GL1*
*rpk1-6* allele:								
*N2995*XPIN1GFP_1	71	-	14	-	15	-	1 [1 %]	*GL1/GL1*
*N2995*XPIN1GFP_2	314	-	18	-	32	-	0 [0 %]	*GL1/GL1*
*N2995*XPIN1GFP_3	223	-	32	-	69	-	11[3.3 %]	*GL1/GL1*

^a^ presence(+) or absence (−) of trichomes indicated

^b^ approx. % of all seedlings

### SAM-less monocot seedlings are capable of developing ectopic meristems on the cotyledon

**Fig. 2:**
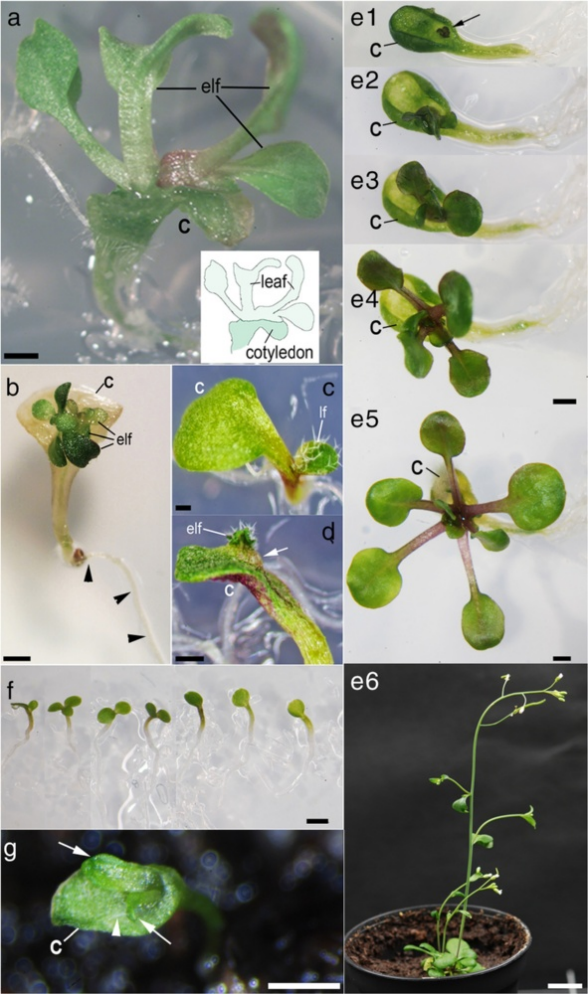
SAM-less *rpk1* seedlings produce ectopic shoot meristems on cotyledons. **a** Monocot *rpk1-7* seedling with an adaxial ectopic shoot meristem (eSM) carrying several leaves (inset: scheme for clarification). **b** The same on a *rpk1-7* monocot seedling from long-term cultivation. The cotyledon has lost its greening. Arrowheads point to the root. **c-d** Monocot *rpk1-7* seedlings in *GL1* background with a normally positioned SAM **c** and with an eSM **d** respectively. Note the trichomes on the normal and ectopic primary leaves. A characteristic tissue outgrowth carries the eSM (arrow). **e1-e5** Growth of an eSM (black arrow) on a cotyledon from a *rpk1-7* seedling during the first two weeks. **e6** The same after one month. **f** Progeny from the eSM *rpk1-7* plant shown in **e1-e6**. **g** A *rpk1-6* monocot seedling carrying two leaf outgrowths (arrows) on top of an abnormally thickened cotyledon. The arrowhead points to a trichome. Cotyledons (c), normal (lf) and ectopic leaves (elf) are indicated. Scale bars: 1 mm except in **e6** e6: 1 cm

During the analyses of *rpk1-7* monocots we repeatedly found SAM-less seedlings, which could enter another rare developmental route by developing an eSM on the adaxial surface of the cotyledon (Fig. 2). The eSMs did not develop on any other SAM-bearing dicot or monocot *rpk1* seedling and displayed some specific characteristics. Firstly, the eSM was positioned on the recognizable adaxial not on the abaxial site of the cotyledon (Fig. 2a-e). Secondly, the eSM appeared in median position on the cotyledon i. e. near the mid-rip (Fig. 2a, b, e1-e5). Thirdly, the eSM generated primary leaves with irregular phyllotactic patterns not additional cotyledons (Fig. 2a, b). Primary leaves of the original line carrying the *glabra1* mutation did not form the trichomes. However, back-crossing to *GLABRA1* background (Table 1) demonstrated that these developed the leaf specific trichomes (Fig. 2c, d). The eSMs generated single leaf organs or (in the other extreme) even rosettes with fertile shoots (Fig. 2e6). The resulting pedigree exhibited a similar range of cotyledon defects (Fig. 2f, Additional file 1: Figure S1). A search in *rpk1-6* for a similar ectopic outgrowth revealed not more than one case among 737 seedlings (Fig. 2g) showing that this special structure is significantly rare. In order to assess the frequency of eSMs systematically, we grew large numbers (>10.000) of *rpk1-7* seedlings in another genetic background (Table 2). The average amount of SAM-bearing and SAM-less monocots remained in the known range. However, the occurrence of eSMs was rare, had no predictable frequency in different pedigrees and was always linked to SAM-less monocots. Together, our observations showed that SAM-less monocot seedlings result from different mutations in *RPK1*. Therefore, in the following we concentrated on the analysis of the *rpk1-7* alone.

**Table 2:** Frequency of ectopic meristems (eSMs)

Line^a^	Dicots & others^b^	Monocots + SAM [%]^c^	Monocots -SAM^d^ [%]^c^	Monocots + eSM [%]^c^	Monocots (+SAM, −SAM, +eSM) [%]^c^
*rpk1-7* X *KNAT2:GUS* A	1246	52 [4]	12 [0.9]	0 [0]	4.9
*rpk1-7* X *KNAT2:GUS* B	1902	165 [7.7]	62 [3]	1 [4.5x10^−4^]	10.7
*rpk1-7* X *KNAT2:GUS* C	1995	187 [8.3]	62 [2.8]	1 [4.4x10^−4^]	11.1
*rpk1-7* X *KNAT2:GUS* D	421	49 [10.1]	12 [2.7]	1 [2.0x10^−3^]	12.8
*rpk1-7* X *KNAT2:GUS* E	332	50 [12.9]	5 [1.5]	1 [2.5x10^−3^]	14.4
*rpk1-7* X *KNAT2:GUS* F	3202	262 [7.4]	78 [2.3]	2 [5.6x10^−4^]	9.7

^a^Outcrosses to marker line *KNAT2p:GUS*, repeatedly selfed and with *gl1/gl1* and *non-KNAT2p:GUS* background

^b^Only monocots vs. others were considered, seedlings with irregular cotyledons, e. g. unequally sized (= anisocots), were not separately counted

^c^Percentage of all seedlings counted

^d^In three randomly selected batches tested, between 15-66 % of initial –SAM seedlings developed a late SAM

### The eSM displays organizational similarities to wild-type SAMs

**Fig. 3:**
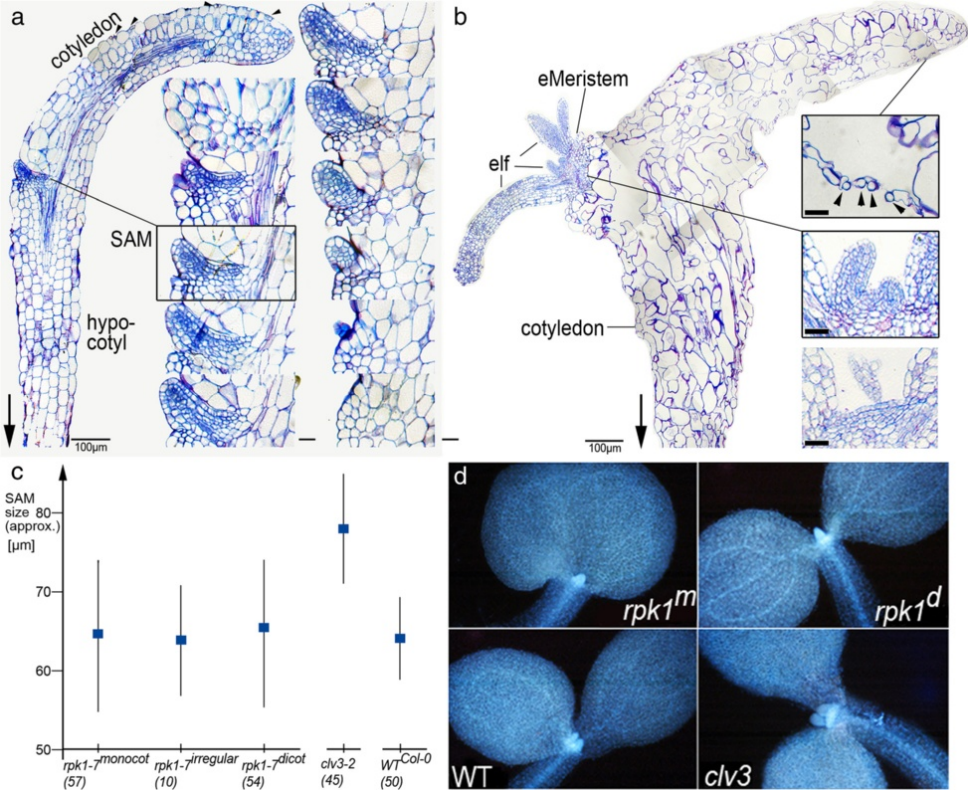
Meristem structure and size of monocot *rpk1-7* seedlings. **a** Median section of a seedling with SAM and insets showing a magnified series of sections through the SAM (that of the median section is framed). Stomata are separated by other epidermal cells (arrowheads). Note the seemingly terminal position of the cotyledon, while the vascular elements demonstrate a lateral origin. **b** An eSM seedling (left). Insets show magnifications with details (right): irregularly spaced stomata (top, arrowheads); a regularly shaped meristem with leaf primordia (middle); a further section few microns apart from the former (bottom). Arrows point towards the root. Scale bars: 100 μm (left parts of **a** and **b**) and 20 μm (insets). **c** Means and SDs of *rpk1-7* seedlings with one, two irregularly sized and two normal cotyledons and of wild-type and the *clv3* mutant respectively (brackets: numbers of seedlings analysed). **d** Representatives of the different seedlings (except irregular seedlings)

A plant with an eSM was histologically compared with a “normal“ monocot seedling (Fig 3). The latter developed a SAM at the base of the cotyledon, which harboured regular cell files belonging to epidermis, palisade, mesophyll and xylem/phloem tissue, very much like a SAM of a dicot seedling. Within all tissues, the cells showed regular cell size proportions and vacuolation. Stomata were found above small cavities and were well separated from each other by epidermal cells (Fig. 3a). The SAM was positioned at the base of the remaining cotyledon where it would be normally expected. Its organization consisted of a group of small densely stained cells, which laterally gave rise to leaf primordia (Fig. 3a). As seen from the vascular system, the origin of the cotyledon is lateral and not terminal.

The cotyledons of SAM-less monocots always displayed an adaxial/abaxial orientation as evidenced by well-developed laminae, their bending, the form of the continuous hypocotyl-cotyledon structure, lacking a real petiole, and the position of the developed SAM (Figs. 1 and 2). However, the tissues and cells were significantly disproportionate in shapes and sizes (Fig. 3b). Abnormal shapes of epidermal cells indicated abnormal (not anticlinal) divisions. Stomata were sometimes neighboured to each other (Fig. 3b, top inset) and inner cells could be extremely large (> > 100 μm in length) and loosely attached to each other. In contrast, the regular (cellular) organization of the eSM was reminiscent of a wild-type SAM or the SAM in monocot siblings (compare Fig. 3a and b). A series of leaf primordia emerged from a cluster of small, plasma rich (densely stained) cells in the centre. The emerging eSM possibly caused a tension along the proximo-distal axis such that the cotyledon bent to form a buckle, which in turn produced a cavity beneath (Fig. 3b, compare with Fig. 2d).

Next, we addressed the question whether the loss of SAM in monocot *rpk1-7* is the extreme of a gradual reduction of meristem size. Due to the abundance of plasma, shoot apical meristem cells of DAPI-stained seedlings show intensive fluorescence, which can be taken as an approximation to meristem size [25]. SAMs of seedling phenotypes of *rpk1-7* (i. e. dicots, monocots, seedlings with irregular e. g. fused cotyledons) were compared with wild-type SAMs (Col-0 ecotype) as well as with mutant *clavata3* SAMs (Fig. 3c, d). The latter have been shown to be significantly larger than wild-type SAMs [26]. SAM-less monocot seedlings did not show densely stained SAM cell clusters (not shown). The distribution of SAM sizes of *rpk1-7* seedlings significantly overlapped with the sizes of wild-type SAMs. In contrast, the control *clavata3* mutant exhibited significantly larger SAMs (Fig. 3c, d). We conclude that the representatives of the different *rpk1-7* cotyledon variants are not members of a continuum of gradual decrease of SAM size. This suggests that the SAM-less monocot phenotype results from the incapability to reach a threshold required to establish a SAM (e. g. a critical amount or activity of coordinated SAM gene expression).

### Cotyledons of SAM-less monocot *rpk1-7* seedlings display SAM-specific gene expression

**Fig. 4:**
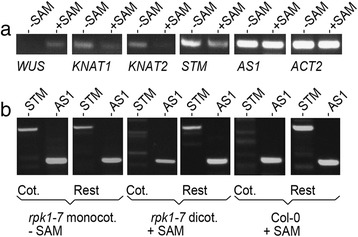
RT-PCR analysis of monocot *rpk1-7* seedlings with and without SAM. **a** Analysis of complete seedlings with (+ SAM) and without (− SAM) shoot meristem. RT-PCR amplification products after 40 cycles with primer pairs of genes as indicated. Note, that the expression of *KNAT1* and *2* was present but very weak in seedlings with SAM. **b** Analysis of *rpk1-7* monocot (− SAM) and *rpk1-7* and wild-type dicot seedlings (+ SAM) separated into cotyledon tissue (Cot.) and (epi- and) hypocotyl and root tissue respectively (Rest)

Next we analysed expression of SAM-specific genes such as *WUS, STM, KNAT1* and *KNAT2* (Fig. 4a) by semi-quantitative RT-PCR (see Methods). In this and other experiments care was taken that SAM-less seedlings were in fact devoid of a recognizable (late) SAM and that experiments with separated cotyledon tissue were not contaminated with hypocotyl and root tissue (see Methods). The cotyledon and leaf specific *AS1* [27, 28] was included as control (in addition to *ACT2*). In one experiment, two seedlings of the SAM-less and two of the SAM-bearing group were separately analysed (including those shown in Fig. 1a to 1c). SAM-less seedlings expressed three of the four SAM-specific genes together with *AS1*, which was strongly expressed (Fig. 4a). While *WUS* was not found in these SAM-less seedlings, *STM, KNAT1* and *KNAT2* appeared to be aberrantly expressed in comparison to monocot seedlings with SAMs (Fig. 4a). The aliquots of both *AS1* and *ACT2* displayed significantly stronger expression since these genes have an overall expression in the cotyledon and the rest of the seedling respectively. Testing *STM* and *AS1* (and *AS2*, not shown) in pools of cotyledons separated from the rest of the body, showed *STM* expression in cotyledons of SAM-less seedlings but not in those of controls (Fig. 4b). In addition, *STM* expression was also found in the rest of SAM-less monocots and as expected in the two controls (Fig. 4b). All bands had the expected sizes (as derived from the known transcripts). Additionally, representative bands were sequence verified. The expression of *STM* in both groups of monocot seedlings was comparable. A similar result was obtained using material of single seedlings (Additional file 1: Figure S2).

### In situ hybridization analysis of late monocot *rpk1-7* embryos detects a rare ectopic *STM* expression

We monitored the expression of SAM-specific (*STM, CLV3*) and cotyledon-specific (*PID, ENP*) genes, which starts at very early embryo stages. However, in contrast to our former study [22] we concentrated on late embryo stages for two reasons. First, in late embryogenesis, *PID* and *ENP* show an additional expression in the SAM (e. g. [5]). Second, we wanted to increase the probability to find the expectedly rare ectopic expression of one of these genes in the monocot embryos, which have themselves a rare penetrance.

**Fig. 5:**
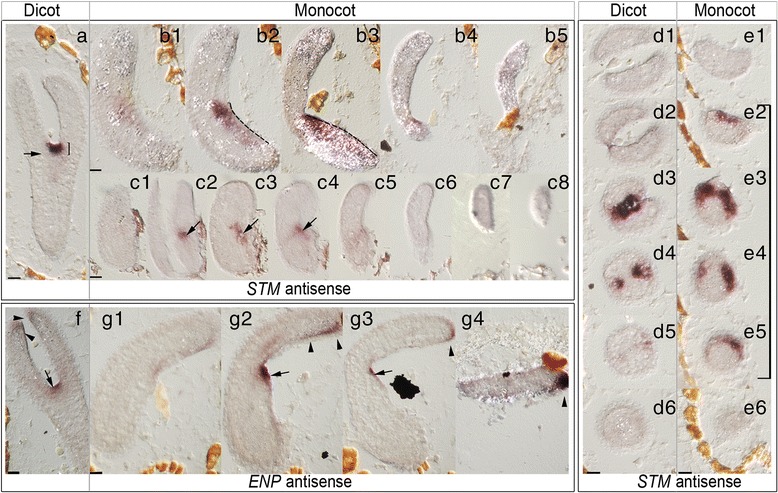
In situ hybridization analysis of monocot *rpk1-7* seedlings. Shown are (serial) sections of torpedo embryos (dicot and monocot embryos indicated). **a–e6** In situ hybridization with the *STM* probe. **f-g4** In situ hybridization with the *ENP* probe for comparison. **a-c8** and **f-g4** show longitudinal sections and **d1-6**; **e1-6** show cross-sections respectively. Brackets in **a** and **d2-5**, **e2-5** indicate the distance of 15-20 μm (cross sections have 3,5 μm thickness). The stippled line in **b2** and **b3** indicates the extension of the *STM* signal along the SAM region and the hypocotyl. Arrows point to the localized SAM signals of *STM* and (late) *ENP* respectively. Arrowheads indicate the additional *ENP* signal in the cotyledon epidermis. Scale bars: 20 μm

Late monocot *rpk1* embryos displayed a “banana“-like appearance with a more or less recognizable notch harbouring the presumptive SAM region. As expected, we mostly detected correct expression patterns. *STM* showed a larger while *CLV3* exhibited a small expression domain as known (Fig. 5a-e6, Additional file 1: Figures S3 and S4 for comparison). Similarly, *ENP* and *PID* showed normal late expression in cotyledons and the SAM (Fig. 5f, g1-g4; Additional file 1: Figures S5 and S6 for comparison). Although any of these probes could have potentially detected an abnormal expression pattern, we found only one among 30 monocots (out of 328 *rpk1-7* torpedo embryos). Considering the 10 % frequency of SAM-less seedlings among monocot *rpk1-7* seedlings, this is in the same range. Surprisingly, in the identified monocot embryo the hybridization with the *STM* probe extended almost along the complete embryonic hypocotyl but not into the cotyledon tissue, with the strongest concentration being at the normal SAM position (Fig. 5b1-b5; stippled line in B2 and B3). The size of the domain expressing *STM* in this specimen clearly exceeded 15-20 μm in apical-basal axis, which is the size displayed in dicot and monocot SAM-bearing *rpk1-7* torpedo embryos (Fig. 5a, d1-d6, e1-e6; brackets). This result coincides with one of the subsequently observed *KNAT2p:GUS* expression pattern variants in SAM-less monocot *rpk1-7* seedlings (see below).

### The SAM-specific *KNAT2p-GUS* activity is variable and abnormally distributed in SAM-less *rpk1-7* monocot seedlings

**Fig. 6:**
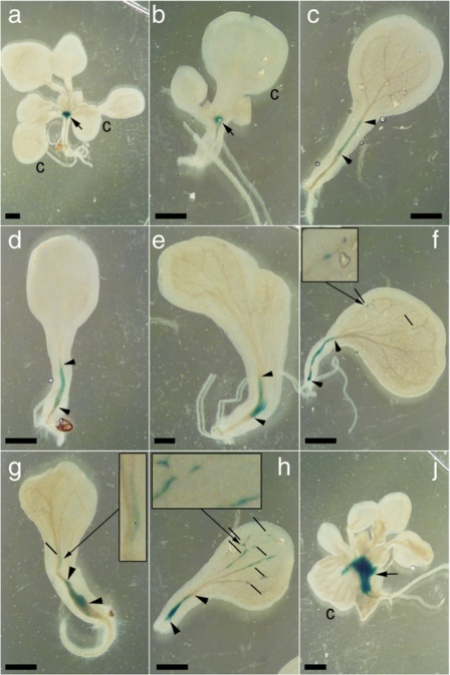
Analysis of *KNAT2p:GUS* reporter construct in *rpk1-7* background. Shown are wild-type **a** and monocot *rpk1-7* **b** with GUS stain in the SAM (arrow), SAM-less monocot *rpk1-7* seedlings **c-j** with weak GUS expression (**c**), with variably extended GUS expression in the presumptive SAM position (**d-h**; arrowheads) and the cotyledon (**f-h**; short lines) and with an eSM in the cotyledon **j**. Insets show details as magnifications. C: marks cotyledon in **a-c** and **j**. Scale bars: 1 mm

In order to obtain a larger number of specimen with informative ectopic expression patterns of a SAM-related gene, we analysed *Arabidopsis* seedlings carrying a *KNAT2p:GUS* reporter [29]. *KNAT2* is a *STM*-dependent transcription factor whose expression is localised in the SAM [30] (Fig. 6a). The monocot pedigree of a *rpk1-7 X KNAT2p:GUS* cross contained normal dicot, SAM-bearing monocot and SAM-less monocot seedlings. The former two exhibited GUS stain as expected at the apex next to the base of the cotyledon(s) (Fig. 6a, b). The SAM-less monocots displayed a spectrum of variants with respect to *KNAT2* expression. Many seedlings showed very weak (Fig. 6c) to more intensive GUS expression in the central (vascular) tissue in the fused hypocotyl-cotyledon structure. This could extend either in direction towards the cotyledon tip or towards the root tip (Fig. 6d-h). The variability was further increased by some seedlings, which displayed smaller or larger patches of GUS staining in the cotyledon lamina (Fig. 6f-h). Monocot seedlings generating an eSM showed a strong GUS staining in the cotyledon (Fig. 6j). The variable *KNAT2* expression in the cotyledon coincided with the results of the foregoing experiments. Thus, all expression data together suggest that SAM-less seedlings display an aberrant SAM gene expression pattern causing the generation of an eSM to be a rare event because it requires the concerted and precise coordination of several SAM genes.

## Discussion

The timely and spatially stochastic alteration of cell division and PIN1 polarity in the embryo epidermis of *rpk1* mutants causes a variable development of the cotyledon primordia, in particular the complete loss of one cotyledon indicating an early developmental accident during globular embryo stages [22]. Later we detected that among monocots of different *rpk1* alleles the loss of the SAM had a low but consistent frequency and seemed to occur together with the generation of a continuous hypocotyl-cotyledon organ lacking a discernable epicotyl region. In this study we have systematically analysed this particular phenotype. Since the SAM-less phenotype is not a specialty of a single allele, we have focussed on *rpk1-7* when analysing the cellular morphology and gene expression patterns.

### SAM-less *rpk1* seedlings lack a recognizable organ separation and display a compromised cell differentiation when developing eSMs

The apex in *Arabidopsis* is formed through antagonistic activities of SAM-specific versus cotyledon/leaf specific genes [31]. Essentially, in the apex *STM* activates *KNAT1/BP* and *KNAT2* (and *KNAT6*) directly or indirectly through repression of *AS1* and *AS2* [32, 33]. Conversely, a complex of the proteins AS1 (a MYB protein) and AS2 (a LOB domain protein), which recruits chromatin-remodeling factors, excludes the activity of SAM specific class I KNOX genes, in particular *KNAT1/BP* and *KNAT2* in leaf and cotyledon tissue [27, 28, 34, 35]. Thus, with the exception of plants, which have exploited the reactivation of SAM-related genes in order to generate compound leaves [36], SAM gene activities are excluded from leaf tissue.

In cotyledon tissue of SAM-less *rpk1-7* seedlings, we detected ectopic expression of the SAM-related *STM, KNAT1* and *KNAT2* genes together with cotyledon specific expression of *AS1*. This means that, antagonistic gene activities were detected within close neighbourhood in the same tissue and likely compromised cotyledon organization by generating tissues and cells with altered position, size and shape as evidenced from histological analysis. Similar profound changes in cell morphology have been observed in leaf tissue ectopically expressing single SAM specific genes (e. g. [37]). In accordance with the defect in PIN1 polarity, the disturbed vascular tissue pattern pointed to an auxin defect. Interestingly, eSMs generated rosettes with irregular phyllotactic patterns. In this context it is worth mentioning, that a balanced homeostasis of auxin and cytokinin impact on shoot development and phyllotaxis [38–40]. The development of a fused hypocotyl-cotyledon organ, at the expense of a petiole connecting hypocotyl and cotyledon, indicated severe perturbations of normal cell differentiation. In spite of these cellular disruptions, the morphology of this fused hypocotyl-cotyledon organ clearly retained the wild-type ab- and adaxial polarity in both *rpk1-6* and *rpk1-7* SAM-less monocots. No radialisation as reported for mutants of adaxial vs. abaxial identity genes was observed [41].

### SAM loss and eSM gain in monocot *rpk1-7* seedlings is likely due to timely and spatially non co-ordinated expression of SAM specific genes

Previous studies showed that, although ectopic (over-) expression of (single) KNOX genes could lead to ectopic SAMs, their stabilization required the balanced and concerted activity of stem cell identity and other SAM genes [30, 37, 42, 43]. Our study shows that this is a main problem in SAM-less *rpk1-7* mutants since the analyzed genes often exhibited a non-coordinated and unbalanced activity. For instance, in one case *WUS* was not expressed in cotyledons of SAM-less monocots while *STM, KNAT1* and *KNAT2* were. The latter also seemed to be even more strongly expressed in the mutant than in the wild-type. Since *WUS* expression is required for SAM generation on first place [44], this explains why these seedlings lacked a shoot meristem in spite of expressing other SAM related genes. Additionally, we detected inconsistencies of expression with respect to space and timing. Seedlings with late SAMs indicated a time-delayed co-ordination. This was also corroborated by SAM-less seedlings, which revealed ectopic *KNAT2p:GUS* signals while others were almost devoid of this activity. The former also showed a spatial defect since GUS staining could occur in quite different positions and with variable extension. These observations explain why eSMs are rare and have no predictable frequency. They only develop by coincidence when all required SAM related genes are active in a concerted fashion and surpass critical values. Similarly, SAMs in “normal“ monocot seedlings overlapped in size with wild-type SAMs instead of showing a continuum of gradually decreasing sizes until reaching a SAM-less seedling.

### SAM-less *rpk1* seedlings are caused rather by lack of positional information than suppression of SAM specific gene activity

The *rpk1* phenotypes raise the question whether RPK1 induces the initiation of cotyledon primordia and the SAM through direct control of the corresponding genes. Both possibilities can be excluded. First, in case of the former, *rpk1* mutants should provide seedlings precisely lacking both cotyledons like *pid enp* double mutants [4]. This has not been the case among all analysed *rpk1* homozygous progenies (> > 10.000). Interestingly, monocot *rpk1* embryos develop only one primordium but establish both cotyledon anlagen [22]. This is compatible with former fate-mapping experiments, which suggest a sequential generation of cotyledons [45]. Second, our data also exclude the possibility that *RPK1* directly controls SAM gene expression and development because SAM-less *rpk1-7* seedlings retain the capacity to express a variety of SAM-specific genes and even to generate eSMs. This corroborates the notion that cotyledons and SAM are largely developmentally independent.

**Fig. 7:**
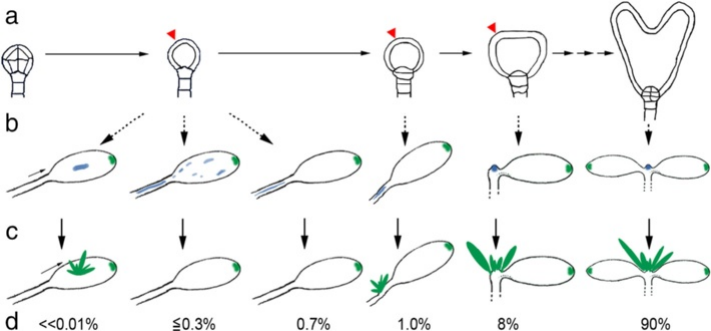
Model explaining early *rpk1-7* defects. **a** The failure to achieve sufficient RPK1 function (red arrowheads) by redundant genes in the *rpk1-7* mutant is stochastic with respect to time and space. Early alterations have more severe effects than late ones on SAM- vs. cotyledon organizing cell groups. **b** Given are possible expression patterns (blue) of *KNAT2* *p* *:GUS* as an example for a SAM-related gene. **c** The realization and maintenance of a shoot meristem depends on the precisely localized and concerted expression of all required SAM genes. **d** Shown are the frequencies of mono- and dicots with and without SAMs/eSMs based mainly on *rpk1-7* data (for details see text and Methods). Green spots symbolize auxin maxima. Note that repeated PIN1 polarity and cell division disturbance can cause additional maxima and lobed cotyledons (see [22])

However, what then causes ectopic SAM gene expression and eSM development? Homozygous *rpk1* mutants differ from previous examples where ectopic shoot meristems were induced in transgenic and complex dominant mutation backgrounds respectively [30, 37, 42, 43, 46]. In contrast, *rpk1* mutants represent a loss-of-function state and form late SAMs at correct positions or eSMs ectopically on top of cotyledons. The *rpk1-7* ectopic shoots, although larger, are reminiscent of epiphyllous inflorescences on foliage leaves in *fil-5 yab3-1* mutants [47] and of ectopic leaf buds in *as1* mutants [27]. However, none of these genes is mutated in *rpk1* plants. The only link to ectopic SAM gene expression (and eSMs) in these mutants is the altered hypocotyl-cotyledon fusion morphology. The probability that eSMs occurred exclusively in morphologically altered SAM-less monocots (6 in 10000; Table 2) just by chance is extremely low (≤10^−12^). This leads us to a model, which integrates the primary defects of *rpk1* mutants, i. e. disturbance of epidermal PIN1 polarity and cell division, and their phenotypes (Fig. 7). In fact, disturbance of PIN1 polarity and auxin homeostasis respectively have been demonstrated to affect initiation of shoot regeneration [39, 48, 49]. Our model takes into account, that due to functional redundancy these defects stochastically scatter along the complete embryo development (Fig. 7). The earlier the *rpk1* defects manifest the more severe are the consequences. The extreme is a fused hypocotyl-cotyledon morphology with the loss of the SAM, which is one of the earliest cell commitments in the embryo (Fig. 7). Apparently, the continuous hypocotyl-cotyledon morphology is accompanied by a loss of positional information because post-embryonically a shoot meristem can form at different positions (late SAMs, eSMs). This circumstance is also reflected in variable ectopic SAM gene expression patterns in those SAM-less monocots, which fail to form a shoot meristem (Fig. 7).

## Conclusions

This study shows that RPK1 does not primarily control SAM genes, even the extreme *rpk1-7* phenotype retains the capacity to resume shoot meristem development (eSM) and to generate a fully functional plant. However, RPK1 does well impact through its primary defects on the generation of shoots and (cotyledon) organs demonstrating a significant extent of morphological plasticity. This plasticity leads to intriguing similarities with extant angiosperms in particular real monocots and monocotyledonous dicots of the genera *Monophyllea* [50] and *Streptocarpus* [51] respectively. *RPK1* mutants are also instructive in a way that sheds light on an aspect that has received less attention. This is the penetrance problem. In contrast to full penetrance of cotyledon-loss in *pid enp* [4], known single or combined mutations in *Arabidopsis*, do not stably produce 100 % monocots [22, 23]. This phenomenon has been previously addressed by studying modifier genes of cotyledon number in *Antirrhinum majus* (e. g. [52]). More recently, an association study using *A. thaliana* ecotypes has identified *RPK1* as an essential (but not the only) gene for shoot organ regeneration [53]. Thus, the *rpk1* monocot phenotype furthers our understanding of angiosperm development in two ways. First, it points to the organizational and genetic peculiarities required to generate a monocotyledonous plant from a dicot. Second, it shows, that it might be promising to search for those genes whose functions have to be altered in concert to obtain full penetrance of monocotyly.

## Methods

### Plant strains and growth conditions

The Col-0 ecotype was used as wild-type reference. The strong *rpk1-7* allele originated from the selfing of a fast neutron mutagenized seed of Col/*gl-1* background and represents an inversion mutation [22]. Monocot *rpk1-6* and *rpk1-7* seedlings were analysed in the original line and in different backgrounds resulting after crossing with different (reporter) lines. In *rpk1-7*, the *gl-1* background results in loss of trichomes characteristic for post-embryonic leaves. Therefore, *rpk1-7* was crossed to *GL-1* background (harbouring the *PIN1p:PIN1::GFP* reporter). The *KNAT2p::GUS* reporter [29] was crossed with *rpk1-7* in order to detect ectopic SAM gene-related expression patterns. Segregating *gl1/gl1* pedigree of this cross lacking the *KNAT2p::GUS* reporter was used for assessing eSM frequency. The *rpk1-6* allele is a T-DNA insertion 357 bp from the ATG in the ecotype WS-2 obtained from NASC (Nottingham Arabidopsis Stock Center; for further details see [22]). This allele was either analysed as original line or as line harbouring the *PIN1p:PIN1::GFP* reporter. Growing of seedlings on soil was essentially as described [22]. Seeds were surface sterilized in calcium hypochloride (ca. 5 %, 15 min) and then washed 3X in H_2_O. Sterile culturing of SAM-less monocot seedlings was initially performed on 0.5X MS in petri dishes and later in magenta boxes respectively under continuous light and 21 °C.

### Microscopy

Semi-thin sections and whole mount analysis of embryos and seedlings were carried out as previously described [4, 12, 54]. Photographs were taken using a ZEISS Axiophot1 microscope equipped with a Digital Nikon camera (F5SLR) and corresponding software (Nikon Camera Control Pro). Epifluorescence microscopy on the same Axiophot used a HBO50 UV/Light-source with a DAPI filter system (Zeiss filter set 01, BP365/FT395/LP397).

### GUS-Staining

Staining of seedlings carrying the GUS reporter construct was carried out after fixation by vacuum infiltrating a solution of NaH_2_PO_4_ (pH 7.0) and 1 % Formaldehyde for 10 min in an Eppendorf tube. After placing the tube for 20 min on ice, the fixative was washed off with 50 mM NaH_2_PO_4_ (pH 7.0) and staining was performed as previously described [55]. SAM-less monocot seedlings showing GUS staining were taken to estimate the proportion of SAM-less monocots with ectopic expression in the cotyledon vs. those with expression exclusively in the hypocotyl.

### RT-PCR and PCR

Plant DNA was isolated following conventional protocols. RNA isolation, reverse transcription and PCR were performed according to the supplier’s instructions using a NucleoSpin®-RNA Plant (Macherey-Nagel) or PolyATract-System IV kit (Promega) respectively. Reverse transcription of total RNA with a TaqMan® kit (Applied Biosystems, Roche) included the following steps: 20 min 25 °C followed by 45 min 48 °C and stopped with 5 min at 95 °C. RT-PCR analysis was semi-quantitative; i. e. for probes to be compared the same amount of RNA material was used in the RT reaction and/or amounts of PCR products loaded were adjusted with respect to the *ACT2* reactions. Fig. 4a, Fig. 4b and Additional file 1: Figure S2 show independent experiments because three different seedling batches were used. Especial care was taken using isolated cotyledon tissue by locating the section at safe distance to the hypocotyl-cotyledon fusion region.

The following forward and reverse primer pairs were used (gene and fragment size in parentheses):

5′-GCCCATCATGACATCACATC-3′ and 5′- CTTTAAGCTCTCTATCCTCAGCTTG-3′ (*STM*; 701 bp fragment); 5′-GGCACCGAGCTTGGGCAGAC-3′ and 5′-GAGACGGTTCAGGGGCGGTC-3 (*AS1*; 322 bp); 5′-TCAGAAGAAGAGATTCAAC-3′ and 5′-AGGGCGAACTTCCGATTGG-3′ (*WUS*; 562 bp); 5′-CACCGTCTGTCTCTGCCTCCTCTA-3′and 5′-ATTCCGCCAACGCTACCTTCTCT-3′ (*KNAT1*; 534 bp); GGAGCTGATCCTGAGCTTGATG-3′and 5′-CACCAATCGAGCAACGCTTGTC-3 (*KNAT2*; 380 bp); 5′-TTGTTCCAGCCCTCGTTTGT-3′and 5′-CCTGGACCTGCCTCATCATACT-3′ (*ACT2*; 323 bp). PCR cycles were: 3 min 93 °C, 40X (45 s 93 °C, 60 s 60 °C and 60 s 72 °C), 3 min 72 °C, 3 min 4 °C.

In order to assess correct gene identities some RT-PCR products were sequenced through EUROFINS/MWG services.

### In situ hybridisation analyses

In situ hybridization, assessment of anti- and sense probes and wild-type expression patterns were as previously reported and had been previously confirmed respectively [4, 22]. In contrast to the study of Luichtl et al. [22], we focused on embryos from early torpedo stage onwards.

## Additional file

Additional file 1:
**Figure S1.** Progeny of an eSM of monocot *rpk1-7* plants. **Figure S2**: RT-PCR analysis of single *rpk1-7* monocot seedlings. **Figure S3**: In situ hybridization of dicot *rpk1-7* embryos with a *STM* probe. **Figure S4**: In situ hybridization of dicot and monocot *rpk1-7* embryos with a *CLV3* probe. **Figure S5**: In situ hybridization of dicot *rpk1-7* embryos with an *ENP* probe. **Figure S6**: In situ hybridization of dicot and monocot *rpk1-7* embryos with a *PID* probe.

## Abbreviations

*ACT2*: *ACTIN2*
*AS1*: *ASSYMMETRIC LEAVES1*
*AS2*: *ASSYMMETRIC LEAVES2*
*BP*: *BREVI PEDICELLUS*
*CLV3*: *CLAVATA3*
*clv3*: *clavata3*
Col-0: Columbia-0
*ENP*: *ENHANCER OF PINOID*
*enp*: *enhancer of pinoid*
eSM: ectopic Shoot Meristem
*gl1*: *glabra1*
GUS: beta-Glucuronidase
*KNAT1*: *KNOTTED1-LIKE ARABIDOPSIS THALIANA1*
*KNAT2*: *KNOTTED1-LIKE ARABIDOPSIS THALIANA2*
*LOB*: *Lateral Organ Boundary*
MIPS: D-myo-inositol-3-phosphate synthase
MS: Murashige Skoog
NASC: Nottingham Arabidopsis Stock Center
*NPH3*: *NON-PHOTOTROPIC HYPOCOTYL3*
*PID*: *PINOID*
*pid*: *pinoid*
*PIN1*: *PINFORMED1*
RAM: Root Apical Meristem
*RPK1*: *RECEPTOR-LIKE PROTEIN KINASE1*
*RPK2*: *RECEPTOR-LIKE PROTEIN KINASE2*
SAM: Shoot Apical Meristem
*sic*: *single cotyledon*
*STM*: *SHOOT MERISTEM-LESS*
WS-2: Wassilewskija-2
*WUS*: *WUSCHEL*
