# Supplementary material for: Ectopic shoot meristem generation in monocotyledonous rpk1 mutants is linked to SAM loss and altered seedling morphology
Source: BMC Plant Biol. 2015 Jul 7;15:171. doi: 10.1186/s12870-015-0556-8 (PMC4492102; doi:10.1186/s12870-015-0556-8)
Supplement: Additional file 1: — Figure S1. Progeny of an eSM of monocot rpk1-7 plants. Figure S2: RT-PCR analysis of single rpk1-7 monocot seedlings. Figure S3: In situ hybridization of dicot rpk1-7 embryos with a STM probe. Figure S4: In situ hybridization of dicot and monocot rpk1-7 embryos with a CLV3 probe. Figure S5: In situ hybridization of dicot rpk1-7 embryos with an ENP probe. Figure S6: In situ hybridization of dicot and monocot rpk1-7 embryos with a PID probe. [file 12870_2015_556_MOESM1_ESM.pdf]

Additional file 1 (Figures S1-6).

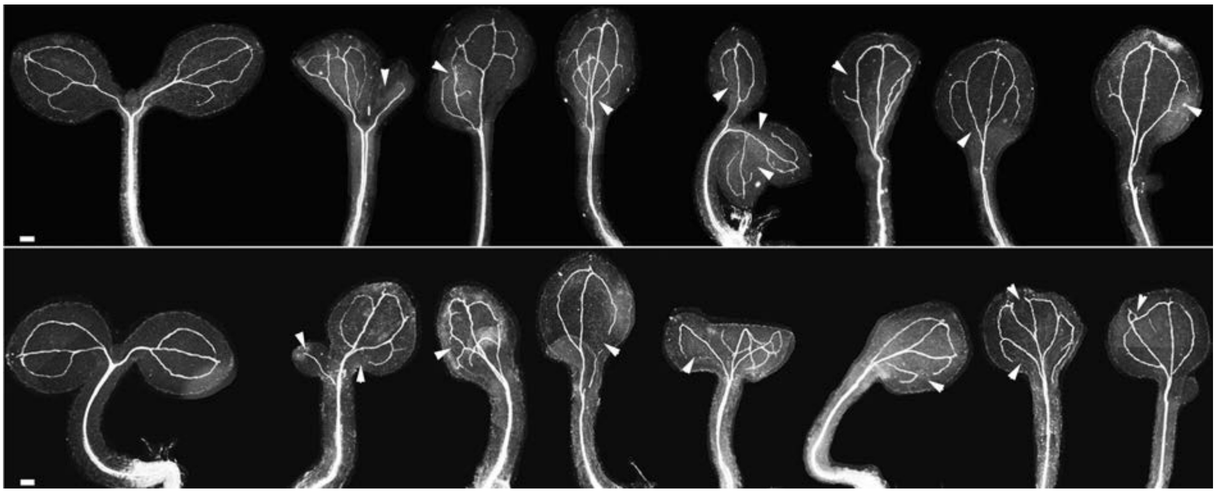

Fig. S1: Progeny of an eSM of monocot *rpk1-7* plants
Shown are Hoyers whole mount preparations of progeny of two different plants (top, bottom), whose aerial organs originated from ectopic shoots in cotyledons. The seedlings developed variable cotyledon defect seedlings, in particular monocot seedlings, with incomplete penetrance. Note the vascular defects (arrowheads). Scale bars: ca. 1 mm.

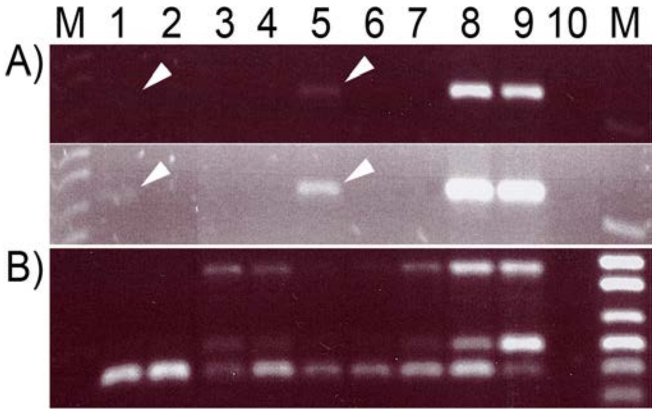

Fig. S2: RT-PCR analysis of single *rpk1-7* monocot seedlings
A) RT-PCR with *STM* primer pair. *STM* positive tissues lead to the amplification of the *STM*-specific band (top). Brightness and contrast have been enhanced in order to visualize weak bands (bottom; arrowheads). B) Shown is a control using an *ACT8* specific primer pair. Note that in different tissues these primers amplify tissue specific bands probably originating from *ACT8* and *ACT8*-homologous sequences of other *ACTIN* genes. For better comparison, controls described in the text were carried out with an *ACT2*-specific primer pair. Material used in the different lanes was of monocot *rpk1-7* seedlings at the same age. 1) Complete SAM-less seedling (1). 2) Cotyledon of a SAM-less seedling (2). 3-5) SAM-less seedling with a small, late developed SAM: Cotyledon (3), the 1st and 2nd postembryonic leaf (4), stem with SAM (5). 6-9) Seedling with a normal SAM: Cotyledon (6), 1st and 2nd postembryonic leaf (7), stem and axils (8), flower buds and apex with SAM (9). 10) Col-0 DNA. M: Size marker.

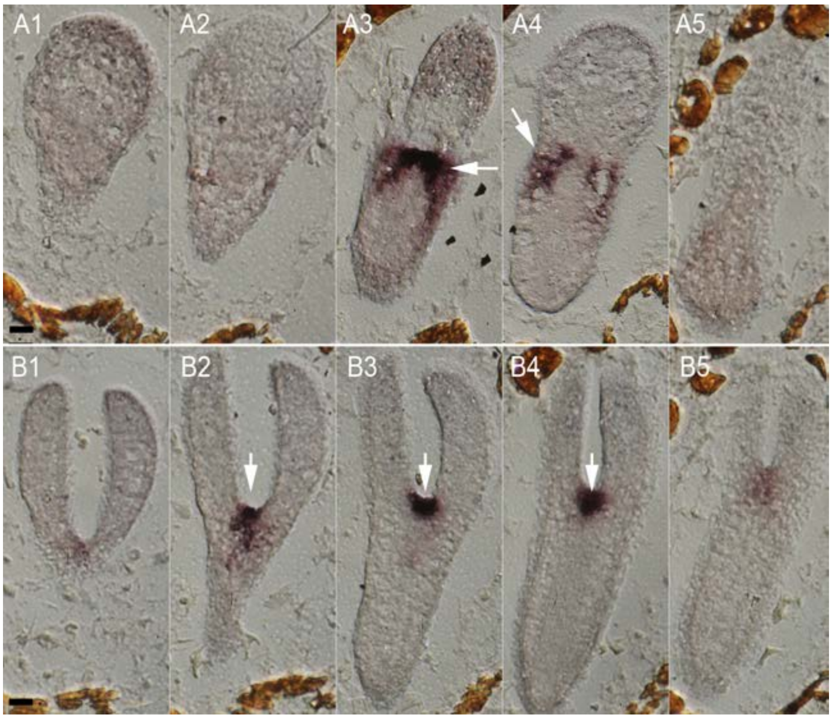

Fig. S3: In situ hybridization of dicot *rpk1-7* embryos with a *STM* probe

A1-A5) Frontal sequence of sections through the embryo: through the first cotyledon, then moving through the apex plateau with the SAM and strong STM signal (arrow) and ending in the second cotyledon. B1-B5) Sequence of lateral sections through the embryo: both cotyledons and the apex plateau with the SAM and strong *STM* signal (arrow) in between are visible. Scale bars: 20µM.

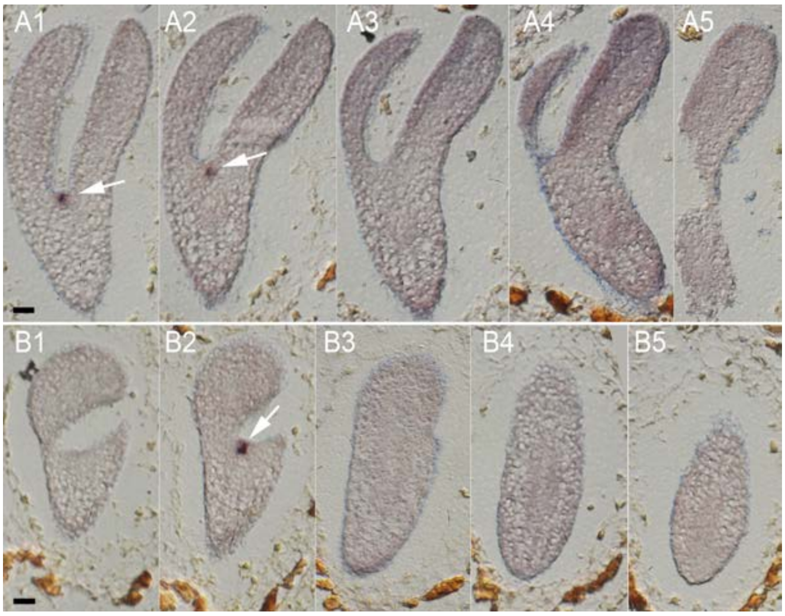

Fig. S4: In situ hybridization of dicot and monocot *rpk1-7* embryos with a *CLV3* probe

A1-A5) Sequence of lateral sections through a dicot embryo: both cotyledons and the apex plateau with a weak *CLV3* signal (arrow) in a small domain of the SAM region are visible. B1-B5) Sequence of lateral sections through a monocot embryo: the single cotyledon and the apex plateau with a weak *CLV3* signal (arrow) in a small domain of the SAM region are visible. Scale bars: 20µM.

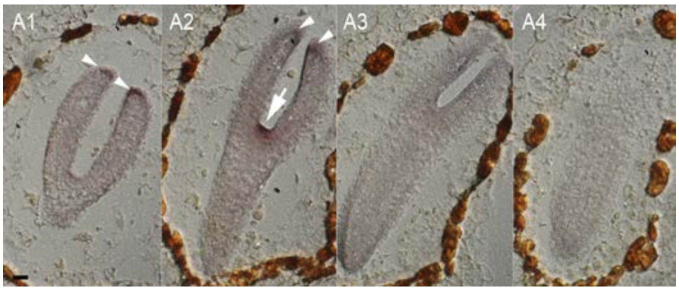

Fig. S5: In situ hybridization of dicot *rpk1-7* embryos with an *ENP* probe
A1-A5, B1-B5) Sequence of lateral sections through a dicot embryo: cotyledons and the apex plateau with an *ENP* signal (arrow) in the SAM region are visible. Note the additional signals at the inner cotyledon sides and the strong signals in the cotyledon tips (arrowheads). The signals in the cotyledon are visible from early on whereas the signal in the SAM appears in torpedo stages. Scale bars: 20µM.

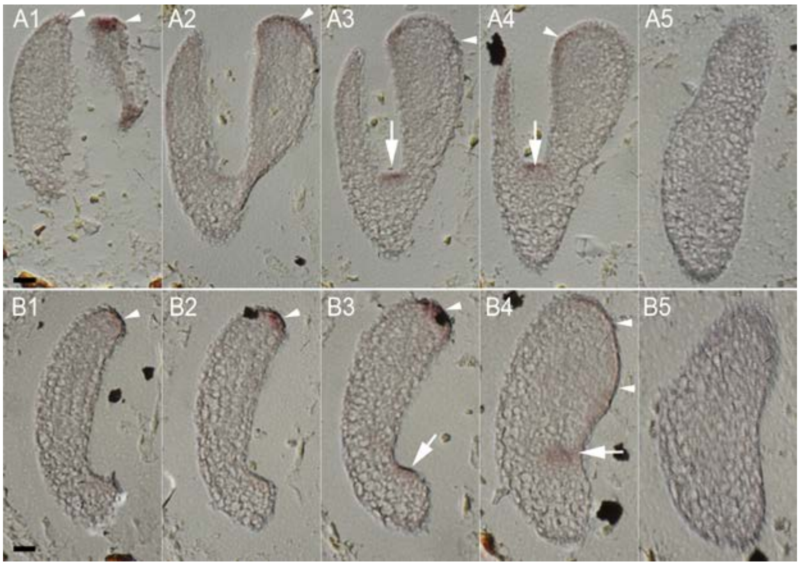

Fig. S6: In situ hybridization of dicot and monocot *rpk1-7* embryos with a *PID* probe
A1-A5) Sequence of lateral sections through a dicot embryo: both cotyledons and the apex plateau with a weak *PID* signal (arrow) in the SAM region are visible. Note also the (weak) signals in the cotyledon tips (arrowheads). B1-B5) Sequence of lateral sections through a monocot embryo. Details as in A1-A5. Scale bars: 20µM.
